# Supplementary material for: Switch between critical percolation modes in city traffic dynamics
Source: Proc Natl Acad Sci U S A. 2018 Dec 27;116(1):23–8. doi: 10.1073/pnas.1801545116 (PMC6320510; doi:10.1073/pnas.1801545116)
Supplement: Supplementary File [file pnas.1801545116.sapp.pdf]

Supplementary Materials for

Switch between critical percolation modes in city traffic dynamics

Guanwen Zeng, Daqing Li*, Shengmin Guo, Liang Gao, Ziyou Gao*, H. Eugene Stanley*, Shlomo Havlin

*correspondence to: daqingl@buaa.edu.cn, zygao@bjtu.edu.cn or hes@bu.edu

www.pnas.org/cgi/doi/10.1073/pnas.1801545115

TABLE OF CONTENTS

Data and Methods **........ 3**

Data description 3

Data preprocessing 3

Supplementary Text **........ 5**

Percolation process in urban traffic 5

Critical threshold and the giant component size of traffic percolation 7

Critical percolation modes in Shenzhen 9

Effective long-range connections 11

Results for city with rare highways 12

Effective dimension with highways 13

References **........ 15**

Data and Methods

Data description

As a megacity with population over 20 million, Beijing, the capital of China, is an ideal system for traffic research. The central area of Beijing (within the 5th Ring Road of Beijing) covers over 700 km^2 land, with a road network containing over 50,000 roads as network links and close to 27,000 intersections as network nodes. The velocity dataset covers real-time velocity records of road segments in Oct. 2015, including a representative holiday period in China, the National Day (from Oct. 1st to Oct. 7th). Velocity (km/h) is recorded using GPS devices in floating cars (e.g. taxies and private cars), with resolution of 1 minute. The road network in Shenzhen includes about 22,000 links and 12,000 nodes, with the same type of velocity dataset as that of Beijing.

Data preprocessing

In real traffic networks, roads can be classified into different states according to their velocity level. A road is considered as functional only if its velocity level meets a given demand. We set a variable threshold q ($0 \leqslant q \leqslant 1$) to denote the velocity demand of drivers at a given time. We sort the velocity data measured during a day for each road segment in increasing order, and choose the 95 percentile as the standard maximal value of this road v_{ij}^m. Then we normalize the velocity $v_{ij}(t)$ of each road

from site i to site j at time t, and get the relative velocity, $r_{ij}(t)= v_{ij}(t)/ v_{ij}^m$. Note that $v_{ij}(t)$ is usually different from $v_{ji}(t)$ due to the direction of the traffic flow. For traffic state in each road segment, we compare its relative velocity $r_{ij}(t)$ with the given threshold q. If $r_{ij}(t) \geqslant q$, the road segment meets the demand and is considered functional; otherwise, the road segment is dysfunctional and removed from the original road network.

In this way, we can construct a functional network of the traffic dynamics from the original topology for any given q. Obviously, for q=0, every road segment satisfies the demand and the functional traffic network is the same as the original road network. On the other hand, if q=1, the functional network becomes completely fragmented as almost all the links are removed from the original network. Hence, we can study the percolation process of how the global functional network of traffic flow disintegrates into local clusters of traffic flows as the demand level increases. As q increases from 0 to 1, the functional traffic network becomes diluted, which can be regarded as "traffic percolation" (1). In traffic percolation, there exists a critical threshold value of q at each time, denoted by $q_c(t)$, where the giant component of the dynamical network breaks down into fragmented local traffic clusters and the second largest cluster reaches its maximum (2, 3).

Supplementary Text

Percolation process in urban traffic

In Fig. S1 we demonstrate the percolation process where the giant component of high-speed traffic flow shows a phase transition with increasing q. At $q_c(t)$ the second largest cluster shows a maximum. We also demonstrate in Fig. S1*E* and Fig. S1*F*, the structure of the giant component near criticality. The traffic percolation threshold varies at different instants. As seen from Fig. S1*A* and Fig. S1*B*, the value of $q_c(t)$ in a workday (Oct. 15th, 2015) is lower than that of a representative holiday (Oct. 1st, 2015) at a morning rush-hour instant (8:00AM). However, at noon the results of the two days seem very similar, as seen in Fig. S1*C* and Fig. S1*D*. According to the definition, $q_c(t)$ reflects the percolation critical point of the global dynamical traffic. If a car is traveling with relative velocity above $q_c(t)$, it will be trapped in local isolated clusters (Fig. S1*F*). Thus $q_c(t)$ is a measure of the maximal relative velocity that one can travel around a major part of the city (i.e. the giant component of the functional traffic network), which reflects the global efficiency of the city traffic at a given time.

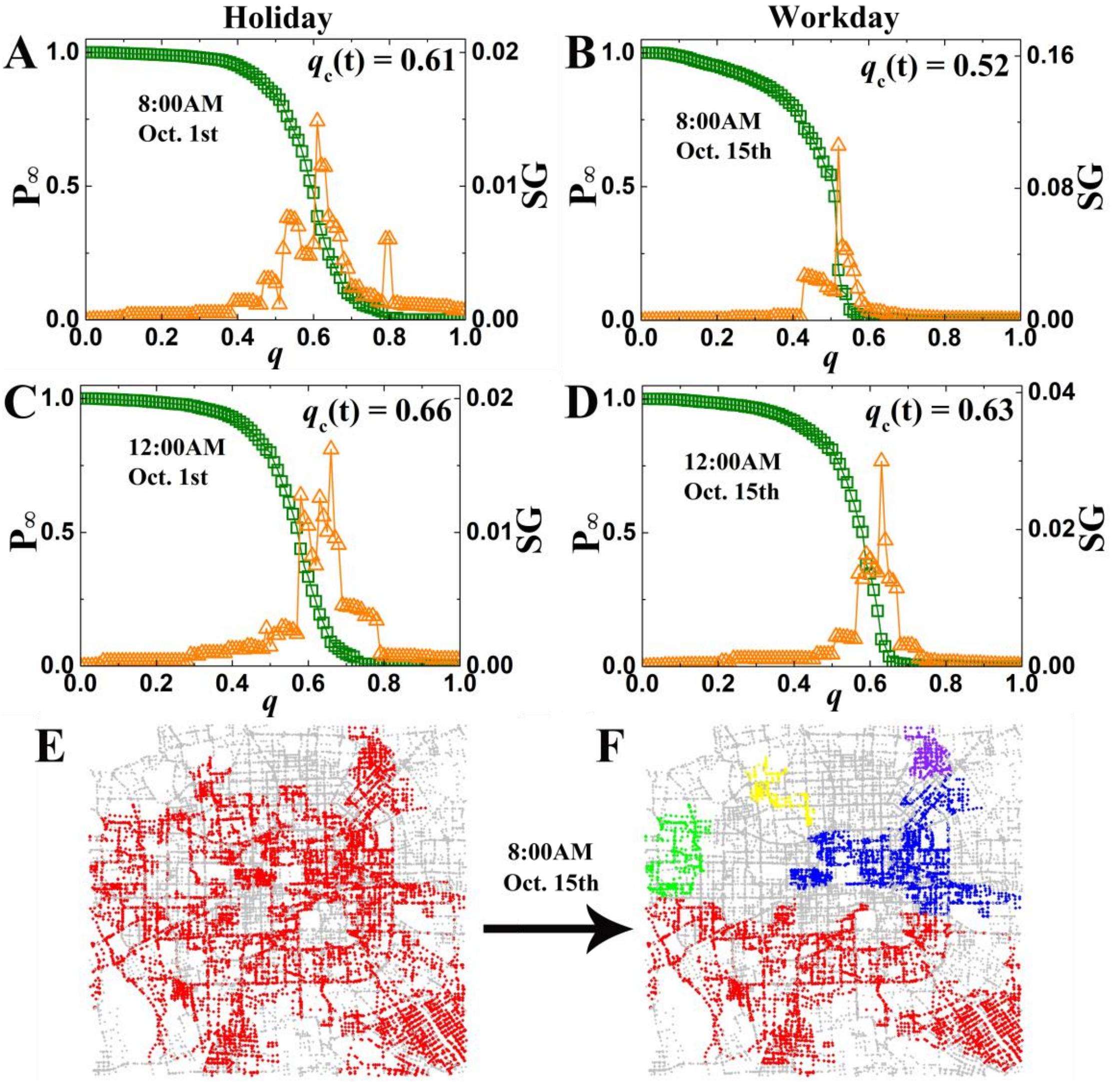

Fig. S1

Percolation processes in urban traffic at different instants on (*A*)(*C*) Oct. 1st (holiday), and (*B*)(*D*) Oct. 15th (workday). As q increases, the giant component (□) of traffic flows decreases and the second largest cluster (△) shows a maximal value at criticality $q_c(t)$ as expected in percolation transition. Note that the quantities are rescaled in the figure. (*E*)(*F*) A typical example of the traffic percolation transition (*E*) below and (*F*) above the critical threshold is shown.

Critical threshold and the giant component size of traffic percolation

Next, we study how $q_c(t)$ evolves with time during a day. Fig. S2*A* shows that $q_c(t)$ is significantly different at rush hours between days off (including holidays and weekends) and workdays. That is, $q_c(t)$ in working days is much smaller than in days off during rush hours, whereas they are similar during other periods. This can be also validated by the distribution of $q_c(t)$ during rush hours in both periods in Fig. S2*B*, which shows that the distributions of $q_c(t)$ are well separated. With the above results, we can conclude that during rush hours the percolation efficiency of the city traffic in workdays is significantly lower than that in days off. This has been further confirmed in Fig. S2*C* and Fig. S2*D* that the giant component size between days off and workdays only has difference during two rush hours, suggesting the possible two percolation modes for city traffic.

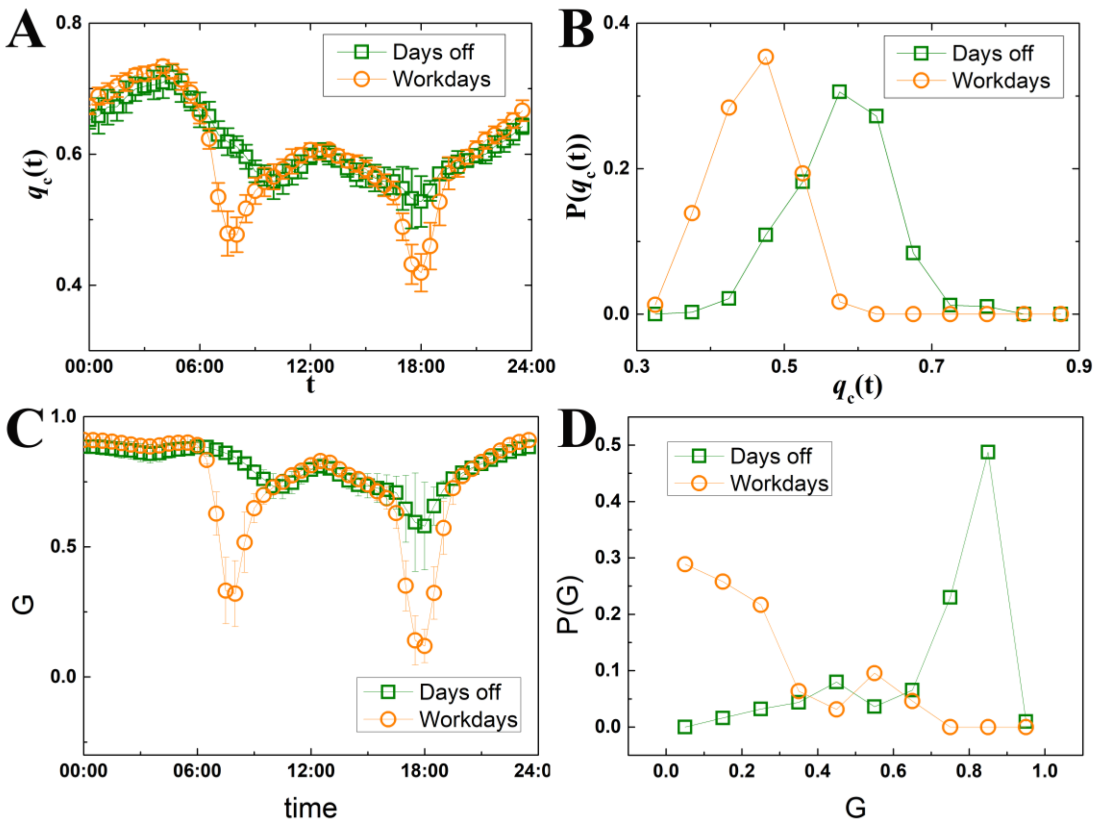

Fig. S2

Critical threshold and the giant component size of traffic percolation in Beijing. (*A*) Values of $q_c(t)$ during a day for days off (■) and workdays (○) respectively, with the resolution of 30 minutes. (*B*) Distribution of $q_c(t)$ during rush hours in days off (■) and workdays (○). Rush hours here mean 7:30~8:30AM and 17:30~18:30PM. (*C*) Size of the giant component (G) in a day during days off (■) and workdays (○) respectively, for percolation threshold q = 0.5, with the resolution of 30 minutes. (*D*) Distribution of G during rush hours in days off (■) and workdays (○). Rush hours here mean 7:30~8:30AM and 17:30~18:30PM.

Critical percolation modes in Shenzhen

Besides Beijing, we have also analyzed the critical percolation properties of Shenzhen, which is one of the largest cities in South China. Similar to the results of Beijing, values of $q_c(t)$ in Shenzhen (Fig. S3*A*) are well separated during rush hours in days off and working days, and the giant component in Fig. S3*B* breaks earlier at a rush-hour instant in a workday than that in a holiday.

We also find in Shenzhen (Fig. S3*C*) that the percolation critical exponent can be classified into mainly two modes between rush hour in workdays and other periods. This is explained by the distribution difference of velocity on city expressways in Fig. S3*D*. Note that the upper bound of critical exponents in Fig. S3*C* is lower than that in Beijing, which is probably due to the spatial features of static road network in different cities.

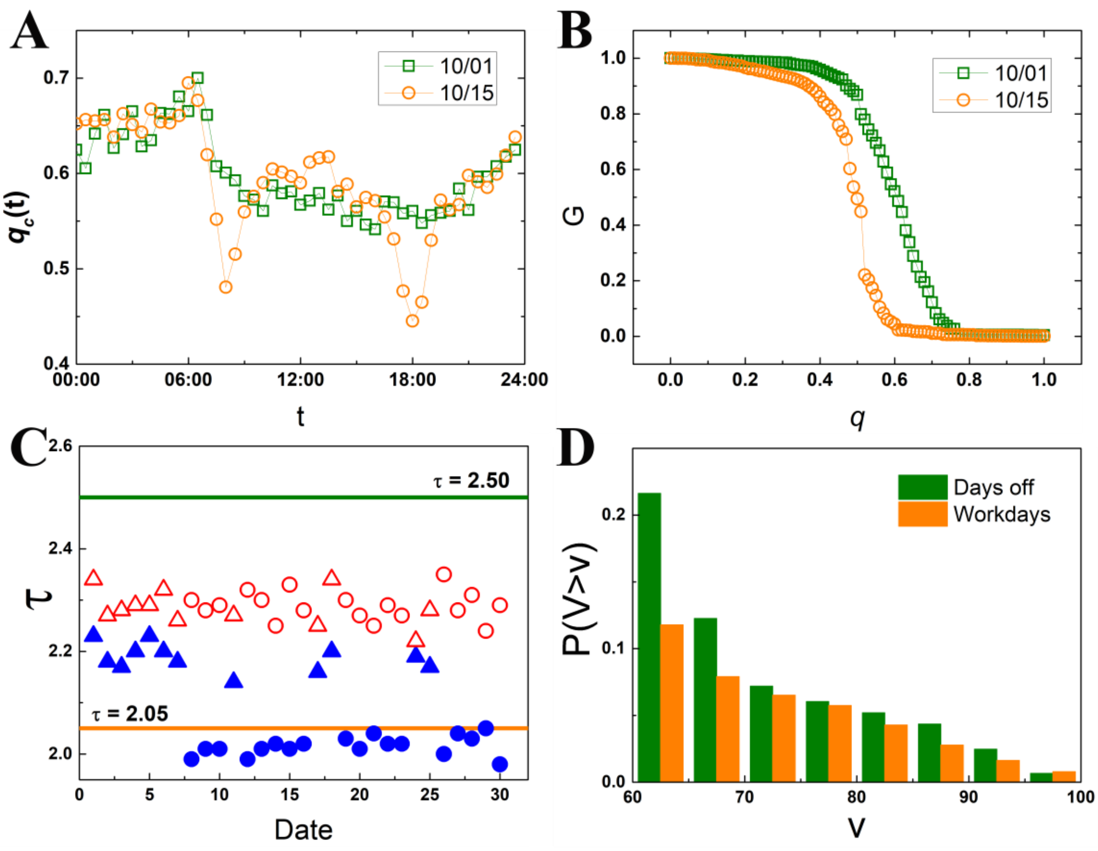

Fig. S3

(*A*) The critical percolation threshold, $q_c(t)$, as a function of time during a typical holiday (Oct. 1st) and workday (Oct. 15th). (*B*) Percolation process of city traffic in Shenzhen at a rush-hour time (8:00AM) in a typical holiday (Oct. 1st) and workday (Oct. 15th). G is the size of the giant high-speed traffic component. (*C*) Critical percolation exponents of cluster size distribution in Shenzhen. Values of τ in specific periods of every day, including: rush hours in days off (▲), rush hours in workdays (●), non-rush hours in days off (△) and non-rush hours in workdays (○). Rush hours here mean 7:30~8:30AM and 17:30~18:30PM, while non-rush hours are 11:00AM~13:00PM every day. The theoretical results of mean field for small-world ($\tau = 2.50$) and two-dimensional lattice percolation ($\tau = 2.05$) are also marked as horizontal lines. (*D*) Cumulative velocity distribution of highways in days off (olive)

and workdays (orange), in the traffic network. We only focus on velocities larger than 60 km/h.

Effective long-range connections

To explain our findings for different percolation critical exponents, we here study the properties of high-speed highway roads, which can form effectively long-range connections. In Fig. S4*A*, it is seen that the number of high-speed highways with velocity larger than 70 km/h in Beijing decreases to a lower level during rush hours in both days off and workdays. However, during days off there are always more high-speed highways than that during working days, especially on the rush-hour periods (i.e., between 7:30AM and 8:30AM), as in Fig. S4*A*. To identify the long-range connections that significantly influence the transition of the city traffic, we compare the high-speed highways effectively switched on and off during different time periods. It is seen in Fig. S4*B* that the high-speed highways, appearing during days off while disappearing during working days, can form effectively long-range connections. These roads are usually the ring roads of highway as the city arterials, whose congestion will result in the breakdown of the global city traffic.

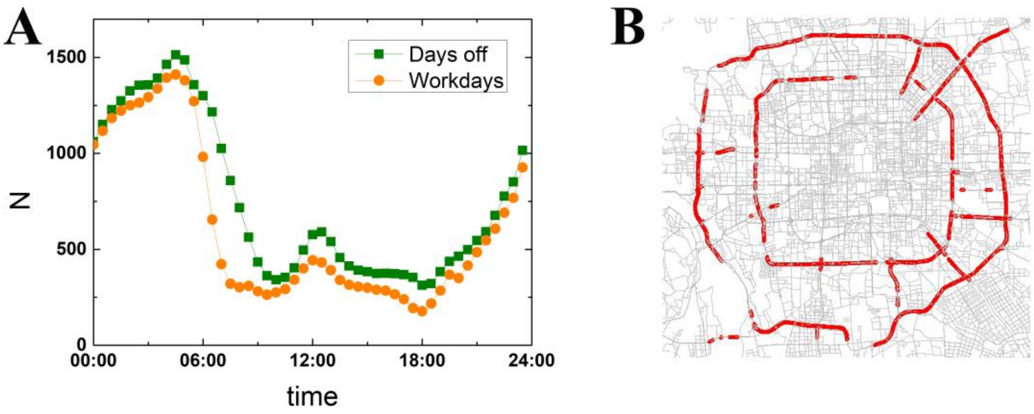

Fig. S4

Effective long-range connections in urban traffic. (*A*) The number of high-speed highway roads (with velocity larger than 70 km/h) evolves with time during different periods: days off (squares) and working days (circles). (*B*) The high-speed highways have velocity smaller than 70 km/h during working days, while having higher velocity (above 70 km/h) during days off (marked as red segments).

Results for city with rare highways

Here we also identified an example of a small city Jinan in China, which has only few urban highways. When comparing the highway structures between Beijing and Jinan, we see in Fig. S5*B* that highways in Jinan are mainly located at the edge of the city, differing from the mesh-like shape of urban highways in Beijing. Accordingly, we observe in Fig. S5*A* only one type of percolation close to two-dimensional case. Indeed, no clear switch found in megacities including Beijing is found here in this small city. However, we expect that with the increasing urbanization, highways will be constructed in more and more cities, and our findings may help regulators to

understand more their relevant impact.

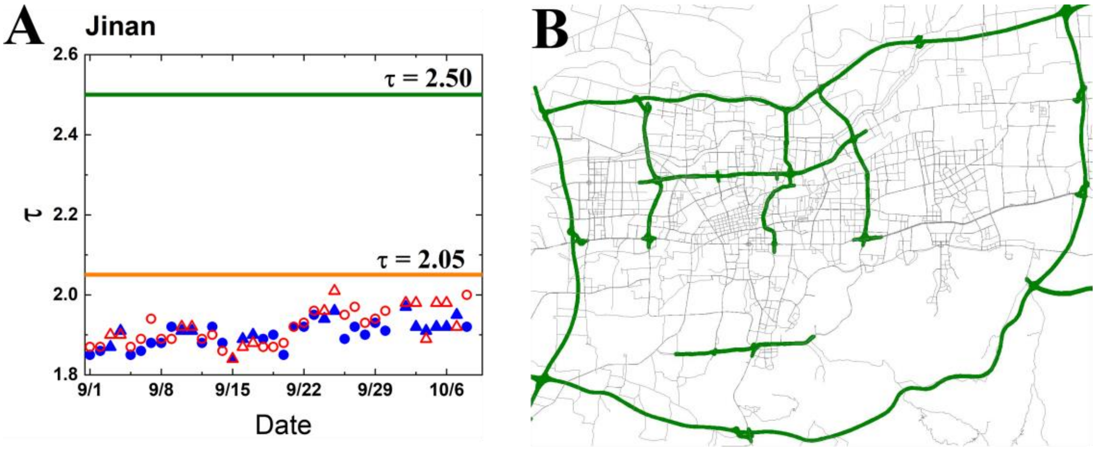

Fig. S5

Situations in a city with few urban highways. (A) Critical exponents for the cluster size distribution of city traffic in Jinan. Values of τ in specific periods of every day, including: rush hours in days off (▲), rush hours in workdays (●), non-rush hours in days off (△) and non-rush hours in workdays (○). Rush hours here mean 7:30~8:30AM and 17:30~18:30PM, while non-rush hours are 11:00AM~13:00PM every day. The theoretical results of high dimensional mean field for small-world (τ=2.50) and two-dimensional lattice percolation (τ=2.05) are also marked as horizontal lines. (B) Highways in Jinan. The roads colored in green are highways.

<u>Effective dimension with highways</u>

Highway can actually act 'effectively' like shortcuts, due to their advantage of velocity as link weight. Thus, highways 'connect' two distinct nodes with much shorter travel time comparatively, instead of direct connection by structural shortcuts

in the networks. Therefore, we test the reachable area as a function of travel time, which reflects 'effective dimension' considering the real traffic. As shown in Fig. S6 below, while this effective dimension is close to 2 when highways have similar velocity as other roads, it becomes much larger with the higher velocity of highway.

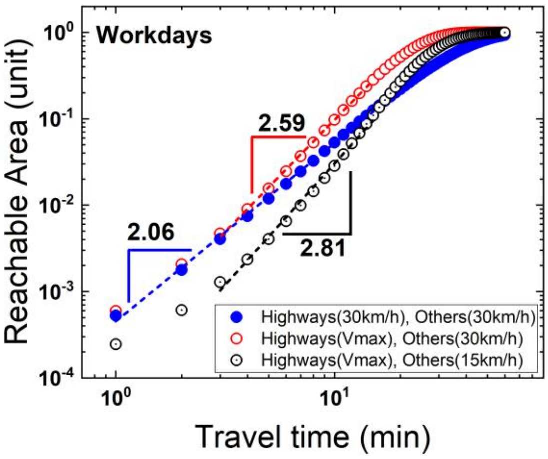

Fig. S6

The reachable area within a given time from a given site. Here other roads have velocity of 30km/h or 15km/h, while highways have maximal velocity for free flow and 30km/h for congestion.

References

1. Li D, *et al.* (2015) Percolation transition in dynamical traffic network with evolving critical bottlenecks. *Proceedings of the National Academy of Sciences* 112(3):669-672.
2. Bunde A & Havlin S (1991) *Fractals and disordered systems* (Springer).
3. Stauffer D & Aharony A (1994) *Introduction to percolation theory* (CRC press).
